# On the Periodic Discharge of Ova, and the Function of Menstruation

**Published:** 1845-08

**Authors:** 


*On the Periodic Discharge of Ova, and the Function of Menstruation.*—The following propositions embody the most important conclusions that have been formed by the best authorities on this highly curious subject.

1. Menstruation commences at the period of the *maturity* of the ovules. 2. The final cessation of the catamenial secretion coincides with the abolition of the formative function of the germs. 3. The ovaries of women, who have ceased to menstruate, never contain the appearance of any vesicles that have recently burst, or that are about to do so (Negrier.) 4. At each menstrual period, the highly excited state of the ovaries induces in the female a decided propension to coition. 5. The aptitude for fecundation is greater on those days that immediately precede the menstrual discharge. 6. In all the lower animals, the ovaria become tumid during the season of rutting. 7. Women, in whom there is a congenital absence of the ovaria, never truly menstruate, however perfect may be the structure of the uterus and other parts of the generative system. 8. The extirpation of these organs puts a complete stop to menstruation,in cases where this function had been already established. 9. Women, in whom there is a congenital absence of the uterus, but in whom the ovaria are normally developed, experience every month all the phenomena of menstruation, the sanguineous discharge alone excepted. 10. The catamenial secretion ceases completely in women, in whom the ovaria have become affected with organic degeneration. 11. It has been asserted by some writers that lascivious girls have—in this respect like the common hen—occasionally discharged ova from the vagina, and that mere voluptuous thought will suffice *pour ebranler* these minute vesicles. 12. In very many women, the menstrual period is preceded by severe colicy pains, attributable most likely to the turgid and excited state of the ovaries. 13. In those who suffer much at these periods, the cavity of the uterus sometimes becomes lined with a soft flocky membrane—a genuine *membrana caduca*—the formation of which is entirely independent of coition. 14. Lastly, in that singular case of monstrosity—in which the two girls, Helen and Judith, were united to each other by the posterior and lower parts of the back—the catamenial discharge took place in different quantities and at different times from each subject, although there was a complete anastomosis between the abdominal vessels of the two.—*Ibid. from Mem. pour ser. à l'et. des Mal. des Ovaires, par Achille Chereau.*

